# Heterogeneous biocatalysts based on porous polymer and lipase from *Aspergillus niger* obtained from SSC employing agroindustrial residues as raw material

**DOI:** 10.1007/s13205-026-05042-0

**Published:** 2026-09-09

**Authors:** Tatiane de Souza Ribeiro, Ezaine Cristina Corrêa Torquato, Evelin Andrade Manoel, Eliane Pereira Cipolatti, Luciana da Cunha Costa, Gizele Cardoso Fontes Sant’Ana

**Affiliations:** 1 Programa de Pós-Graduação em Engenharia Química, Universidade do Estado do Rio de Janeiro (UERJ), Rio de Janeiro, RJ Brazil; 2 Departamento de Farmácia, Faculdade de Ciências Biológicas e Saúde, Universidade do Estado do Rio de Janeiro, Rio de Janeiro, RJ Brazil; 3 Departamento de Bioquímica, Instituto de Química, Universidade Federal do Rio de Janeiro (UFRJ), Rio de Janeiro, RJ Brazil; 4 Departamento de Engenharia Química, Instituto de Tecnologia, Universidade Federal Rural do Rio de Janeiro (UFRRJ), Seropédica, RJ Brazil

**Keywords:** Filamentous fungi, Castor oil, Enzyme immobilization, Fermentation, Lipase production, Solid-State Cultivation

## Abstract

This study aimed to produce extracellular lipases from Aspergillus niger using solid-state cultivation (SSC) of cottonseed bran and a blend of cottonseed bran and wheat bran (agroindustrial coproducts) as low-cost substrates. The highest activity (93.1 U/g _dry mass_) was achieved with cottonseed bran supplemented with castor oil at a moisture content of 54%. A mesoporous poly(styrene-co-divinylbenzene) (poly(Sty-co-DVB)) support (surface area of 259 m^2^ g^−1^) was used for lipase immobilization. The immobilization yield ranged from 67.3% to 98.2%, whereas the biocatalyst's Ra was much lower, ranging from 5 to 30%. The heterogeneous biocatalysts were evaluated for the hydrolysis of castor oil, achieving ester-to-free fatty acid conversions ranging from 17.7% to 45%. Their performance was compared with that of commercial immobilized lipases (Lipozyme TL, Lipozyme RM, and Lipozyme 435). The SSC-derived biocatalysts showed activity comparable to the commercial ones. Furthermore, the heterogeneous biocatalyst maintained its catalytic efficiency over five consecutive hydrolysis reactions, with no significant decrease in conversion. Overall, this study demonstrates the feasibility of using agro-industrial by-products as inexpensive substrates for lipase production and highlights the potential of SSC-derived immobilized biocatalysts as sustainable, cost-effective alternatives for enzymatic hydrolysis.

## Introduction

Enzyme-catalyzed processes have gained considerable prominence due to their efficiency under mild reaction conditions, high selectivity, and stability. Furthermore, they enable the production of high-purity products while reducing environmental impact, thereby representing a sustainable alternative to traditional chemical catalysts (Cipolatti et al. 2019). Lipases (triacylglycerol ester hydrolases, EC 3.1.1.3) are among the most versatile industrial biocatalysts, catalyzing not only the hydrolysis of triglycerides, their natural substrates, but also in a variety of other reactions, including esterification and transesterification. This broad catalytic repertoire makes lipases highly attractive for numerous industrial applications, including food, pharmaceutical, oleochemical, and biofuel sectors (Sampaio et al. 2022). However, the economic feasibility of the enzyme-based bioprocesses remains strongly influenced by the costs associated with the enzyme production (Medeiros et al. 2023).

Strains of the filamentous fungus *Aspergillus niger* are widely recognized as efficient producers of extracellular lipases. In addition, *A. niger* has been granted as “Generally Recognized industrial as Safe” (GRAS) (Ohara et al. 2018). The thermostable, 1,3-specific lipase produced by *A. niger* is notable for its stability, selectivity, and tolerance to glycerol, which confer competitive advantages in biotransformations. This enzyme represents a promising option for applications, particularly in the food and chemical sectors, where it has been successfully employed for triglyceride modification, structured lipid synthesis and biodiesel production (Feng et al. 2020; Xu et al. 2024).

Solid-state cultivation (SSC) is characterized by the growth of microorganisms on a moist solid substrate in the absence or near absence of free water. The solid matrix simultaneously serves as both a nutrient source and a physical support for microbial colonization and development (Perwez and Al Asheh 2025; Khaswal et al. 2024). SSC is considered an efficient platform for lipase production, distinguished by its high yield and productivity, low contamination risk, simple bioreactor requirements, and lower production costs (Medeiros et al. 2023). Additionally, the SSC simulates the natural habitat of filamentous fungi, favoring hyphal growth, metabolic activity, and the secretion of extracellular enzymes, thereby enhancing lipase production.

The use of agro-industrial co-products in SSC contributes to sustainability and economic viability of the processes by serving as an inexpensive source of carbon, nitrogen, minerals, other nutrients, and moisture often requiring minimal or no pretreatment (Ohara et al. 2018; Perwez and Al Asheh 2025). The selection of an appropriate substrate is a key factor for efficient lipase production, it is essential that these co-products contain a significant lipid content, as the type of lipid used as a carbon source is the main factor influencing enzymatic activity expression (Carvalho et al. 2023; Pereira et al. 2019). Accordingly, several lipid-rich substrates have been employed as lipase inducers in SSC, including soybean oil (Kreling et al. 2021), olive oil (Putri et al. 2020; Medeiros et al. 2023), sesame oil, jatropha oil (Putri et al. 2020), and tilapia oil (Medeiros et al. 2023).

The use of various agro-industrial by-products promotes sustainability by enabling proper waste management and adding value to agricultural residues (Brazilian Agricultural Research Corporation (EMBRAPA 2020)). Several agroindustrial co-products have been used as raw materials for lipase production by *Aspergillus niger*, including wheat bran (Costa et al. 2017; Ohara et al. 2018; El-Ghonemy et al. 2020; Kreling et al. 2021; Medeiros et al. 2023; Ali et al. 2023), cottonseed cake (Nema et al. 2019; Mandari et al. 2020; Medeiros et al. 2023), rice bran (Costa et al. 2017; Putri et al. 2020; Ali et al. 2023), cottonseed meal, and orange peel (Ohara et al. 2018). Other reported substrates include jatropha seed cake (Putri et al. 2020), soybean meal (Costa et al. 2017; Prabaningtyas et al. 2018; Ohara et al. 2018), palm kernel cake (Prabaningtyas et al. 2018), rice husk (Nema et al. 2019), chicken feather meal (Oliveira et al. 2019), red gram husk (Nema et al. 2019; Mandari et al. 2020), sunflower oil cake, wheat germ, olive cake, jojoba cake, cottonseed waste, rice bran and husk, corn cob (El-Ghonemy et al. 2020), guava leaves, peanut husk, corn husk, reed grass (Ali et al. 2023), sugarcane bagasse (Ali et al. 2023; Zambrano et al. 2023), corncob (Kreling et al. 2021), and *Prosopis juliflora* (Mandari et al. 2020). The wide diversity of plant-derived substrates highlights the remarkable versatility of SSC.

Cottonseed meal stands out as an industrial by-product capable of inducing lipase production due to its favorable lipid composition, which is rich in linoleic, oleic, and palmitic acids (Medeiros et al. 2023). According to Ohara et al. (2018) and Aguieiras et al. (2019), cottonseed meal represents a promising support matrix and nutrient source because of its high lipid content, providing a favorable environment for enzyme synthesis. The increasing availability of this agro-industrial co-product further enhances its industrial relevance. In 2024, Brazil surpassed the United States in cotton production, reaching more than 3.7 million tons (Brazil Cotton Producers Association (ABRAPA 2024)).

Wheat bran is another agro-industrial by-product widely recognized as a suitable substrate for SSC due to its low lignin content, high nutritional value, favorable particle structure, and high porosity, all of which promote microbial colonization, oxygen transfer, and enzyme production (El-Ghonemy et al. 2020). According to Arora et al. (2018) and El-Ghonemy et al. (2020),possesses a well-balanced nutritional composition and is can be incorporated as a co-substrate to improve the physical properties of the cultivation medium, particularly by increasing substrate porosity and aeration, thereby enhancing fungal growth and lipase production. Moreover, its widespread availability and low cost make wheat bran an attractive feedstock for large-scale SSC processes. Currently, Brazil ranks 14th in global wheat production, with an estimated yield of 10.3 million tons for the 2023/2024 harvest (National Supply Company (CONAB 2024)).

The use of cottonseed meal and wheat bran as carbon sources can be justified by their favorable nutritional characteristics, wide availability in Brazil, and economic viability, particularly for lipase production by *A. niger* under SSC. These raw materials not only enhance the efficiency of the fermentation process but also increase the addition by providing a sustainable and cost-effective solution for industrial biotechnology. Despite advances in the use of agro-industrial residues for lipase production by filamentous fungi, few studies have evaluated pure cottonseed meal or its combination with wheat bran as substrates for the solid-state cultivation of *A. niger* 11T53A14, particularly under varying moisture levels.

Castor oil is also an effective lipid-based carbon source for the cultivation of *A. niger*, distinguished by its wide production in Brazil, biodegradability, and cost-effectiveness (Gonçalves et al. 2022). Brazil is the world’s second-largest producer of castor oil, with 87% of the cultivated area concentrated in the state of Bahia (National Supply Company (CONAB 2023)). Dutra et al. (2008) demonstrated that SSC of *A. niger* supplemented with 2% (w/w) castor oil yielded the highest lipase activity (23.7 U/mL), surpassing other oils used as inducers, such as soybean, olive, corn, and palm oils.

Castor oil hydrolysis is one of the main strategies for obtaining ricinoleic acid. Ricinoleic acid ((9Z,12R)-hydroxyoctadecenoic acid) is a high-value hydroxylated unsaturated fatty acid that serves as a key platform molecule for the synthesis of several industrially relevant chemical intermediates, such as sebacic, heptanoic, and undecylenic acids (Xu et al. 2024). Enzymatic hydrolysis of castor oil can be performed under mild conditions. This approach reduces energy consumption, minimizes the degradation of thermolabile compounds and undesired side reactions, making it an environmentally friendly and industrially attractive alternative for ricinoleic acid production. (Gupta 2016; Rathod and Pandit 2009),

The application of free enzymes faces challenges such as inability to be reused. Enzyme immobilization represents an effective strategy for overcoming the limitations associated with free enzymes by enabling efficient biocatalyst recovery and multiple reuse cycles, thereby enhancing operational stability and possibility of substantially reducing process costs. Among the available support materials, poly(styrene-*co*-divinylbenzene) (poly(Sty-*co*-DVB)) has attracted considerable attention for lipase immobilization owing to its hydrophobic nature and the simplicity of particle synthesis, which allows tailoring of physical properties suitable for enzyme immobilization (Torquato et al. 2025; Ribeiro et al. 2022; Pinto et al. 2019, 2014).

The present study aims to evaluate lipase production by *Aspergillus niger* under solid-state cultivation (SSC) using cottonseed bran and wheat bran, with castor oil serving as an inducer of lipase synthesis. In addition, this work seeks to develop a heterogeneous biocatalyst based on lipases produced by SSC and a mesoporous poly(Sty-co-DVB) support, and to conduct a preliminary study of the hydrolysis of castor oil using these heterogeneous biocatalysts.

## Materials and methods

### Lipase production by *A. niger* in Solid State Cultivation (SSC)

#### Preparation of raw materials

Wheat bran and cottonseed meal were kindly provided, respectively, from a local market in Leblon (Rio de Janeiro/RJ, Brazil) and by Embrapa Cotton in Campina Grande (Paraíba, Brazil). Castor oil was also purchased in Campina Grande (Paraíba, Brazil). To serve as a matrix support, the cottonseed meal was comminuted in a knife mill (Marconi®) with a 6 mm sieve.

#### Physicochemical characterization of agro-industrial by-products

Moisture, pH, ash, lipids, and total solids were determined using the methods of the Instituto Adolfo Lutz (2008). The tests were performed in triplicate, and the results were expressed as mean ± standard deviation (SD) in g/100 g.

#### Activation and maintenance of the microorganism

The strain used in this study was *A. niger* 11T53A14, a mutant lineage provided by Embrapa Agroindustry de Alimentos (Rio de Janeiro/RJ, Brazil). The microorganism was maintained in sterilized frozen soil and activated in inclined tubes containing SOCAREAN solid medium, which contained olive oil (2% w/w) as the carbon source and the nutrients described by Couri and Farias (1995). Activation was performed in two successive stages, with incubation of the tubes at 32 °C for 7 days in each stage. After the second activation, approximately 10 mL of sterile Tween 80 solution (0,3% v/v) was added to the tubes to release the conidia. The concentration of conidia in the suspension was determined using a Neubauer chamber, and the inoculum volume was adjusted to achieve a final concentration of 10^7^ conidia per gram of solid medium.

#### Lipase production by *A. niger* in Solid-State Cultivation (SSC)

Lipase production by *A. niger* 11T53A14 was carried out by SSC using 3.4 g of cottonseed meal or a binary mixture with wheat bran (1.7 g each), with or without 2% castor oil as an inducer. The assays were conducted in 100 mL plastic flasks with a diameter of 4 cm, a height of 11 cm, and a base area of 12.57 cm^2^. In these flasks, the agro-industrial by-products were inoculated with 10^7^ spores/g of medium and incubated at 32 °C for 48 h. To evaluate the effect of initial moisture content in the fermentation medium on enzyme production, moisture contents of 30, 45, and 60% (dry basis) were tested. After cultivation, enzyme extracts were obtained by adding 2.4 mL of 0.1 mol L⁻^1^ sodium citrate buffer (pH 5) per gram of medium, followed by shaking in a rotary incubator (170 rpm, 32 °C, 1 h). The soluble extract was collected by vacuum filtration through a Buchner funnel coupled to a Kitassato and nanocellulose membranes (47 mm in diameter, 0.45 μm pore size), yielding solid-free enzymatic solutions for subsequent analyses.

#### Determination of enzyme extract activity

The enzymatic activity of the soluble extract of *A. niger* was determined using *p*-nitrophenyl laurate (*p*-NPL) as the substrate in 25 mM phosphate buffer (pH 7). Reactions were carried out in glass cuvettes containing 2.2 mL of buffer and 0.25 mL of substrate. The reaction was initiated by adding 50 μL of the concentrated enzymatic extract, and absorbance was monitored immediately at 412 nm using a spectrophotometer equipped with a thermostatic bath at 30 °C. All analyses were performed in triplicate. Hydrolytic activity was calculated from the rate of *p*-nitrophenol formation and expressed in units per milliliter (U/mL), according to Eq. 1 (Ribeiro et al. 2022). One enzymatic unit (1 U) was defined as the amount of enzyme required to release 1.0 μmol of product per minute. Activities were also expressed in units per gram of dry mass (U/g), accounting for the mass of the fermentation medium and its moisture content, as described in Eqs. 2 and 3.

1 $${A}_{H}=\frac{\left(\frac{Abs}{min}\right)\cdot {V}_{f}\cdot f}{{V}_{e}}$$

HA: Hydrolytic activity (IU/mL);

(*Abs* /*min*): Angular coefficient of the line obtained during the advancement of the hydrolysis reaction;

V_*f*_ : Final volume of the reaction medium (mL);

*f:* Spectrum factor obtained from the standard curve with *p*-nitrophenol;

*V*_*e*_*:* Volume of concentrated enzyme extract (mL).

2 $${P}_{u}=\frac{{Q}_{mt}. U}{100}$$

3 $${A}_{Lms}=\frac{{A}_{L}. {V}_{t}}{{Q}_{m- {P}_{u}}}$$

where,

*P*_*u*_: Mass of water sample in the crop (g); *Q*_*m*_: Quantity of mass of the fermentation medium sample used for analysis (g); *Q*_*mt*_*:* Total mass used in Solid State Cultivation; *U:* Moisture content of the Solid State Cultivation medium; *A*_*Lms*_: Lipase activity of dry mass (U/g _dry mass_); *A*_*L*_: Lipase activity in U/mL; *V *_*t*_: Volume of buffer solution (mL).

### Synthesis and characterization of polymeric support for enzyme immobilization

#### Synthesis of the poly (Sty-*co*-DVB)

Poly(Sty-co-DVB) was prepared by aqueous suspension polymerization in a 1000 mL round-bottom flask equipped with a reflux condenser, a digital mechanical stirrer, and a tilting propeller. The aqueous phase was prepared by dissolving poly(vinyl alcohol) (0.5% w/v) and sodium chloride (0.5% w/v) in 200 mL of distilled water, and the final volume was adjusted to achieve an aqueous/organic phase ratio of 4:1 (v/v). The organic phase consisted of styrene (Sty) (0.12 mol) and divinylbenzene (DVB) (0.18 mol), with the addition of the initiator azobisisobutyronitrile (AIBN) (1% mol/mol relative to the monomeric mixture) and a porogenic mixture composed of the solvents toluene and n-heptane (20/80% v/v). The volume ratio of the monomeric to porogenic mixtures was 1:1.5 (v/v). The mixture was homogenized before being added to the aqueous phase. The aqueous phase was added to the flask, stirring was set to 230 rpm, and the organic phase was added slowly over 30 min. The reaction was carried out at 80 °C for 10 h under continuous stirring. At the end of the reaction, the copolymer was collected by vacuum filtration and washed sequentially with distilled water, ethanol, and acetone until the filtrate was clear. The polymeric supports were then dried in an oven at 60 °C for 24 h (Ribeiro et al. 2022).

#### Characterization of the support

Poly(Sty-co-DVB) was characterized by optical microscopy (OM), scanning electron microscopy (SEM), nitrogen adsorption (ASAP) for specific surface area, volume, and average pore diameter, and by Fourier transform infrared spectroscopy (FTIR) and thermogravimetric analysis (TGA). OM images were obtained using a Nikon Eclipse E200 microscope and a BEL Photonics IS 500 camera. SEM analysis was performed on a JEOL JSM-6510LV equipped with an EDS Quantax 70 (Bruker) after gold metallization in a BAL-TEC SCD 005 metallizer. The surface area, volume, and pore diameter were determined by N₂ adsorption, with sample pretreatment at 110 °C for 12 h under vacuum. The data were analyzed using the BET equation (surface area) and the BJH method (pore volume and diameter). FTIR analyses were performed in ATR (diamond crystal) mode with a PerkinElmer Frontier spectrophotometer in the range of 4000–400 cm⁻^1^. TGA was conducted on a TA Instruments Q50, with heating from 50 to 800 °C at 10 °C/min under an inert atmosphere.

### Preparation and characterization of the heterogeneous biocatalysts

#### Immobilization of *A. niger* lipase

This study used lipase from *A. niger* obtained by SSC (item 2.1.3) and commercial *A. niger* (Sigma-Aldrich, CAS number 9001-62-1). The immobilization procedure followed previously described methodologies (Ribeiro et al. 2022; Torquato et al. 2025; Cipolatti et al. 2018). Initially, 1 g of the polymeric support was pretreated with 30 mL of ethanol (95%), then washed with 30 mL of distilled water, and finally with sodium phosphate buffer (5 mM, pH 7). The particles were then stored at 4 °C until use.

The enzymatic solution was prepared by diluting 0.5 mL of lipase (produced or commercial) in 9.5 mL of sodium phosphate buffer (5 mM, pH 7), yielding 10 mL, with the initial activity adjusted to 12.5 U/_g support_.

Immobilization was initiated by adding this solution to 0.5 g of pretreated support in a 50 mL Falcon tube, with agitation at 40 rpm on a roller at room temperature. During the process, 150 µL aliquots of the supernatant were collected at 0, 15, 60, and 120 min to analyze the hydrolytic activity of the soluble fraction, using *p*-NPL as the substrate (0.0086 g dissolved in 5 mL of acetonitrile and 5 mL of dimethyl sulfoxide). The hydrolysis reaction was conducted using 2.2 mL of phosphate buffer (25 mM, pH 7), 0.25 mL of the substrate solution, and 50 µL of supernatant. Immobilization was monitored by measuring the formation of *p*-nitrophenol (λ = 412 nm) in a spectrophotometer coupled to a thermostatic bath at 30 °C.

After immobilization, the biocatalysts were filtered, washed with phosphate buffer (5 mM, pH 7.0), dried in a desiccator, and stored at 4 °C. Enzymatic activity was expressed in international units (1 U = 1 μmol of *p*-nitrophenol/min). All analyses were performed in triplicate. The immobilization yield (IY, %) and recovered activity (Ra) were determined according to Ribeiro et al. (2022).

#### Hydrolytic activity of heterogeneous biocatalysts against *p*-nitrophenyl laurate

The hydrolytic activity of the heterogeneous biocatalysts was determined according to adaptations of the methodologies described by Pinto et al. (2019) and Cipolatti et al. (2018), using *p*-nitrophenyl laurate (*p*-NPL) as substrate. The reactions were carried out at 30 °C, with absorbance monitoring at 412 nm, in a spectrophotometer coupled to a thermostatic bath. The reaction mixture consisted of 2.2 mL of sodium phosphate buffer (25 mM, pH 7), 0.25 mL of *p*-NPL, and 0.075 g of biocatalyst. The hydrolytic activity of the biocatalysts were expressed as enzymatic units (U), where 1 U corresponds to the release of 1 µmol of *p*-nitrophenol per minute. The analyses were performed in duplicate.

#### Hydrolytic activity of the heterogeneous biocatalysts toward castor oil

This study was conducted using the heterogeneous biocatalysts produced in this work, the free commercial lipase from *A. niger*, and commercial immobilized lipases. In addition, these reactions were accompanied by tests involving castor oil in contact with water in the presence of the polymer but without the immobilized enzyme, as well as blank tests conducted with the reagents only, to eliminate the possibility of spontaneous substrate hydrolysis in the absence of a catalyst. The commercial immobilized lipases used were Lipozyme 435 (based on *Candida antarctica*), Lipozyme TL (*Thermomyces lanuginosus*), and Lipozyme RM (*Rhizomucor miehei*).

Hydrolysis of castor oil was carried out using 5 g of castor oil, 1.86 mL of 50 mM sodium phosphate buffer (pH 7.0), and 0.15 g of the heterogeneous biocatalysts. Samples (100 μL) were collected after 24 h, centrifuged, and then added to an ethanol:acetone mixture (1:1, v/v) for acidity determination. Titration was performed with NaOH (0.04 mol·L⁻^1^). The formation of ricinoleic acid was quantified by measuring the medium's acidity, as calculated using Eq. 4. The percentage conversion of fatty acid was calculated using Eq. 5. The assays were performed in triplicate.

4 $$Acidity \left(\%\frac{m}{m}\right)=\frac{V. {C}_{NaOH}.{10}^{-3}.{MM}_{AG}. {10}^{2}}{{m}_{amostra}}$$

where,

*V*: volume of NaOH used in the titration (mL); *C*_*NaOH*_: concentration of NaOH (mol L^−1^) (0.04 mol L^−1^); *MM*_*AG*_: molar mass of the fatty acid used (g mol^−1^); *m*: mass of the sample (g).

5 $$Conversion \left(\%\right)=\frac{ \left({Acidity}_{final}-{Acidity}_{initial}\right).100}{{Acidez}_{inicial}}$$

where, Acidity initial: Acidity of aliquots collected at time zero, before the addition of the biocatalyst; Acidity final: Acidity of aliquots collected after 24 h of reaction.

#### Reuse of the biocatalyst in the hydrolysis reaction

The reuse of the biocatalyst biocat(3) was evaluated during the hydrolysis of castor oil to confirm that its catalytic performance was maintained across consecutive reaction cycles. These reactions were carried out under the same conditions described in Sect. “Hydrolytic activity of the heterogeneous biocatalysts toward castor oil”. After each reaction cycle, the biocatalyst was recovered and reused in a new reaction under the same experimental conditions, for a total of five reuse cycles. The hydrolysis conversion was determined by titrating samples of the reaction medium with a 0.04 mol L⁻^1^ NaOH solution to quantify the free fatty acids formed. All experiments were performed in triplicate.

## Results and discussion

### Determination of the proximate composition of cottonseed and wheat bran

Determining the proximate composition of cottonseed and wheat bran was a crucial step in assessing their potential as raw materials for lipase production. The analysis measured moisture, ash, lipid, and total solids content, providing a quantitative basis for evaluating the quality and composition of the brans (see Table 1). This composition is partial and does not include all nutritional components, such as carbohydrates and additional proteins. The selected parameters are considered essential for Solid-State Cultivation (SSC) using the mutant strain of *A. niger* 11T53A14 and castor oil as an inducer.

**Table 1:** Centesimal composition of raw materials: wheat bran and cottonseed bran

Coproducts	Ashes (g/100 g)	Humidity (g/100 g)	Lipids (g/100 g)	Total solids (g/100 g)
Wheat bran	5.60 ± 0.03	9.30 ± 0.04	4.30 ± 0.02	90.70 ± 0.04
Cottonseed meal	3.80 ± 0.00	6.70 ± 0.10	11.20 ± 0.40	93.30 ± 0.10

Wheat bran has a slightly higher ash content (5.60 ± 0.03 g/100 g) than cottonseed meal (3.80 ± 0.00 g/100 g), indicating a greater concentration of essential minerals and nutrients (see Table 1). According to El-Ghonemy et al. (2020), wheat bran's low lignin content and high nutritional value make it beneficial, supporting microbial growth and enzyme production. Moreover, wheat bran has a higher moisture content (9.30 ± 0.04 g/100 g) than cottonseed meal (6.70 ± 0.10 g/100 g). Costa et al. (2017) found that higher moisture in raw materials can reduce porosity, potentially obstructing oxygen flow and increasing the risk of bacterial contamination. However, these effects vary with the raw material’s characteristics and processing conditions. El-Ghonemy et al. (2020) also note that, despite high moisture, wheat bran’s large specific surface area helps counteract adverse moisture effects by enhancing nutrient distribution and creating a more suitable environment for *A. niger* metabolism in the SSC.

Wheat bran has a total solids content of 90.70 ± 0.04 g/100 g, whereas cottonseed meal has a solids content of 93.30 ± 0.10 g/100 g. Medeiros et al. (2023) reported that wheat bran has a crude protein content of 21.97 ± 0.28 g/100 g, which is lower than that of cottonseed meal. Generally, cottonseed meal has a crude protein content of 22.2–56.2 g/100 g and metabolizable energy of 7.4–11.99 MJ/kg (Olukomaiya et al. 2019). The high protein content underscores the importance of cottonseed meal as a vital nutrient source, potentially enhancing lipase production by *A. niger*.

Cottonseed meal has a higher lipid content (11.20 ± 0.40 g/100 g) than wheat bran (4.30 ± 0.02 g/100 g) (Table 1). Studies show that oil-enriched media effectively stimulate lipase production (Ohara et al. 2018), underscoring the positive role of lipids in this process. Cottonseed meal is known for its versatile composition and typically contains high levels of lipids (Olukomaiya et al. 2019). The oil from this by-product mainly consists of triacylglycerols containing linoleic acid (56.9%), oleic acid (16.9%), and palmitic acid (23.3%), which are known to induce lipase production (Medeiros et al. 2023). According to Carvalho et al. (2023), microorganisms such as *A. niger* secrete lipases that hydrolyze triacylglycerols in the medium, releasing fatty acids essential for cellular metabolism. As fatty acid concentrations decline, microorganisms secrete extracellular lipases to break down the remaining lipids, thereby releasing fatty acids needed for metabolism and cell growth.

In the literature, lipase production by *A. niger* during SSC is strongly influenced by the lipid content of the agro-industrial co-products used. Ohara et al. (2018) showed that cultivating *A. niger* in cottonseed meal, which has a lipid content of 8.36%, at 50% humidity for 48 h resulted in a significant enzymatic activity of 323 U/g. In contrast, Aliyah et al. (2016) found that corn bran, despite its high protein content (9.73%), had relatively low *A. niger* lipase activity (3.92 U/mL) due to its low lipid content (2.12%).

Meanwhile, tofu co-products with a lipid content of 10.7% produced higher lipase levels, reaching 8.48 U/mL. Medeiros et al. (2023) studied a mixture of cottonseed cake (lipid content of 7.80 ± 0.80 g/100 g) and wheat bran (lipid content of 3.06 ± 0.09 g/100 g), supplemented with olive oil, tilapia oil, Tween 80, and Triton X. Cultivation with cottonseed meal at a lower ratio (1.5:3.5) resulted in reduced enzymatic activity of 0.091 U/g using *A. niger*. However, Aliyah et al. (2016) also noted that excessive lipid levels can create a two-phase system that hampers oxygen and nutrient transfer to the microorganism, thereby impacting fungal growth. Therefore, the link between lipid content and enzyme activity is complex and influenced by various factors, including the composition of the raw material mix, added carbon sources, and environmental conditions during fermentation (Costa et al. 2017).

### Lipase production from *A. niger* in Solid State Cultivation (SSC)

Filamentous fungi are considered good producers of extracellular lipases, and the processes for extracting and purifying these enzymes are considered simple (Geoffry and Achur 2018). Lipase production was evaluated in two formulations: one made of cottonseed meal and the other a 1:1 w/w mixture of cottonseed meal and wheat bran, with and without castor oil supplementation (2% w/w). Each formulation was tested at different initial moisture levels—30, 45, and 60% w/w—to identify the optimal moisture level for maximizing enzyme production. The experimental results in SSC are shown in Table 2.

**Table 2:** Solid State Cultivation (SSC) parameters and lipase activity across different formulations and humidities, using an inoculum of 10^7^ spores per gram of medium

Exp	Inductor (%)	Cottonseed meal/ Wheat (% m/m)	^a^Initial Hum (%)	^b^Final Hum (%)	^c^Initial pH	^d^Final pH	^e^Lipase activity (U/g _dry mass_)
1	with castor oil (2)	100/00	35	20	5.5	5.7	25.0
2		100/00	40	23	6.4	4.9	40.2
3		100/00	54	29	6.4	5.5	93.1
4		50/50	31	16	6.2	6.1	14.1
5		50/50	47	34	6.3	5.5	25.3
6		50/50	58	47	6.3	5.1	40.0
7	without castor oil	100/00	33	22	6.6	5.0	20.4
8		100/00	43	38	6.3	5.5	33.1
9		100/00	54	50	6.3	5.5	53.0
10		50/50	31	29	6.3	4.6	12.1
11		50/50	46	34	6.2	5.5	20.0
12		50/50	59	56	6.2	5.2	36.9

ᵃInitial Hum: Initial humidity content of the crop before SSC; ᵇFinal Hum: Final humidity content of the crop before SSC; ᶜInitial pH: pH of the medium before fermentation; ᵈFinal pH: pH of the medium after fermentation; ᵉMaximum lipase activity (U/g _dry mass_): Enzymatic activity unit (considering the dry mass of the raw material)

Formulations containing cottonseed meal exhibited higher lipase activity than those based on a mixture of cottonseed meal and wheat bran under the same cultivation conditions (Table 2). The lipase activity obtained using cottonseed meal and castor oil (exp. 1, 2, and 3) was 1.8, 1.6, and 2.3 times higher than that achieved with the mixture of cottonseed meal, wheat, and castor oil (exp. 3, 4, and 5). These data indicate that cottonseed meal, which has a high lipid content (11.2 ± 0.4%) (Table 1), has great potential for enzyme production in SSC processes. According to Carvalho et al. (2023), the lipid content of the raw material is a key factor for lipase efficiency, directly affecting enzymatic activity.

When comparing experiments 1 to 6 (with castor oil) and 7 to 12 (without castor oil), castor oil supplementation was observed to promote lipase production. This effect was more pronounced at an initial moisture content of 54% when using pure cottonseed meal. In experiment 3 (with castor oil), activity was 93.1 U/g_dry mass_, whereas in experiment 9 (without castor oil), activity was 53.0 U/g_dry mass_, yielding a 1.8-fold difference, even though both conditions had an initial moisture content of 54%. At other moisture levels, the differences in activity were less pronounced. These data suggest that the formulation containing cottonseed meal, castor oil, and 54% initial moisture creates an environment conducive to maximum lipase activity.

The increase in the medium's initial moisture content led to higher lipase activity. In experiments 1 to 3, which used cottonseed meal and castor oil, the recorded lipase activities were 25.0, 40.2, and 93.1 U/g_dry mass_ at initial moisture contents of 35, 40, and 54%, respectively. Lipase activity increased 3.7-fold when the initial moisture content was raised from 35 to 54%. For the combination of cottonseed meal and wheat with castor oil (experiments 4 to 6), lipase activities were 14.1, 25.3, and 40.0 U/g_dry mass_ at initial moisture contents of 31, 47, and 58%, respectively. In experiments without castor oil (experiments 7 to 12), similar increases in lipase activity were observed. In experiments 7 to 9 using cottonseed meal, lipase activities were 20.4, 33.1, and 53.0 U/g_dry mass_ at initial moisture levels of 33, 43, and 54%, respectively, representing a 2.6-fold increase as moisture rose from 33 to 54%. In the mixture of cottonseed meal and wheat without castor oil (experiments 10 to 12), lipase activities were 12.1, 20.0, and 36.9 U/g_dry mass_ at initial moisture contents of 31, 46, and 59%, respectively, indicating a 3.0-fold increase in lipase activity.

Using a mixture of cottonseed meal, wheat, and castor oil (experiments 4, 5, and 6) yielded an average moisture retention of 68% during the SSC, exceeding the 56% average retention observed in experiments with pure cottonseed meal and castor oil (experiments 1, 2, and 3). This improved moisture retention can be attributed to fiber content and porosity, particularly in wheat bran. According to Medeiros et al. (2023), wheat bran has a fiber content of 41.6 ± 2.5 g/100 g, higher than the 27.6 ± 0.7 g/100 g found in cottonseed cake. Possibly, the fibrous structure of the raw material mixture created larger porous or interstitial spaces, improving water retention and promoting microbial growth and lipase production. In experiments 5 and 6, which had the highest initial moisture content (47% and 58%) and the lowest moisture loss during fermentation (27.7% and 19%), greater lipase activity was observed in the studied formulations, reaching 25.3 and 40.0 U/g _dry mass_, respectively.

According to Mandari et al. (2020), adequate moisture content enhances nutrient dissolution and transfer, thereby promoting lipase activity. Nema et al. (2019) observed that activity increased as the initial moisture content rose from 55 to 75%, reaching a peak of 28.1 U/g_dry mass_ at 75% moisture after 24 h of SSC with *A. niger* for lipase production using a mixture of rice husk, cottonseed cake, and red gram husk (2:1:1). Mahadik et al. (2002) reported maximum lipase production from *A. niger* at an initial moisture content of 71% using wheat bran.

Excessively high moisture levels, such as 80%, can reduce feedstock porosity and increase the medium's viscosity, thereby inhibiting fungal growth. Conversely, insufficient moisture limits fungal growth by reducing nutrient diffusion and feedstock swelling (Medeiros et al. 2023). In extreme cases, with less than 12% moisture, no microbial growth was observed (Schmidell et al. 2002) because the moisture required for cell growth was absent. These points underscore the importance of accurately controlling moisture content during SSC to promote optimal growth and enzyme production.

It was observed that the initial theoretical moisture levels were not fully achieved in both formulations, possibly due to differences in the water-retention capacity of the fermentation media. The variations in initial and final moisture contents reflect water absorption and evaporation during SSC, which are influenced by the raw material properties and experimental variables. In experiments 1, 2, and 3 using cottonseed meal and castor oil, a greater moisture loss (44%) was observed, likely due to easier evaporation from the substrate's lower water retention and microbial metabolic activity (Schmidell et al. 2002). The mixture of cottonseed meal and wheat bran retained more moisture than pure cottonseed meal. This improved moisture retention can be linked to the fiber content and porosity of the wheat bran. According to Medeiros et al. (2023), wheat bran has a fiber content of 41.6 ± 2.5 g/100 g, whereas cottonseed cake has 27.6 ± 0.7 g/100 g. The fibrous structure of the raw material mixture (cottonseed meal and wheat bran) likely created larger pores and interstitial spaces, thereby enhancing water retention.

The initial pH of pure cottonseed meal ranged from 5.5 to 6.4 and decreased to final values between 4.9 and 5.7, indicating slight acidity. When combined with wheat, the initial pH ranged from 6.2 to 6.3, yielding final pH values between 5.1 and 6.1, also indicating slight acidity (Table 2). These pH changes are likely due not only to increased lipase activity, which hydrolyzes triglycerides in the substrates and releases fatty acids, but also to microbial metabolism, which produces additional acids and helps lower the medium's pH (Krishna 2005; El-Ghonemy et al. 2020). pH can affect enzyme stability by altering electrostatic interactions within their protein structures, thereby impacting secondary and tertiary structures and, consequently, their activity and stability. Studies report that *A. niger* maintains good pH stability (4.0 – 10.0) during lipase production, and higher pH levels (7.5 – 8.0) do not significantly reduce enzyme production (Nema et al. 2019).

Table 3 compiles data from the literature on the correlation between moisture content and initial pH in SSC and lipase activity produced by *A. niger* when using cottonseed meal and wheat bran. The studies examined various raw materials and inducers, with initial moisture ranging from 50 to 75%. In the present study, lipase activity of 93.1 U/g_dry mass_ was achieved in just 48 h of SSC, using *p*-NPL as the substrate. In contrast, El-Ghonemy et al. (2020) recorded an activity of only 4.8 U/g after 144 h of cultivation, using cottonseed as the substrate and *p*-NPP as the substrate. According to Santos et al. (2018), prolonged fermentation times may decrease enzyme production due to nutrient depletion in the medium.

**Table 3:** Data from the literature and this study on lipase production by *A. niger* under various SSC conditions

Raw Material	Inductor	^a^Initial Hum (%)	^b^Initial pH	T (Cº)	Time (h)	^c^Maximum lipase activity (U/g _dry mass_)	Ref
Cottonseed meal	Castor oil	54	6.4	32	48	93.1	This study
Meal: cotton and wheat		58	6.3			40.0	
Husk: rice and red grass and cotton cake (2:1:1)	–	75	6.0	40	24	28.1	Nema et al. 2019
Cotton seed	–	60	7.0	30	144	4.8	El-Ghonemy et al. 2020
Wheat bran						8.6	
Cotton and wheat bran cake (1.5:3.5)	Olive oil, tilapia oil, Tween 80, and Triton X	70	7.0	30	240	0.1	Medeiros et al. 2023
Cottonseed meal	–	50	^d^ND	30	48	323.0	Ohara et al. 2018
Wheat bran and soya bran (1/3:1/3)						319.0	
Prosopis juliflora, red grass bark, and cottonseed cake (6.66:1.66:1.66)	–	70	7.0	30	72	269.8	Mandari et al. 2020
Wheat bran and rice husks (1/2:1/2)	Glycerol	50	4.9	28	168	11.2	Costa et al. 2017
Wheat bran, corn cobs (80:20)	Sugar cane molasses and soybean oil	60	ND	30	96	7.7	Kreling et al. 2021

^a^*Initial Hum *Initial humidity content of the crop before SSC,

^b^*Initial pH* pH of the medium before fermentation,

^c^Maximum lipase activity (U/g _dry mass_): Enzymatic activity unit (considering the dry mass of the raw material),

^d^*ND* Not determined

El-Ghonemy et al. (2020) reported lipase activity of 8.68 U/g using wheat bran with an initial moisture content of 60% as the raw material. Costa et al. (2017) observed lipase activity of 11.2 U/g using a mixture of wheat bran and rice hull (1:1 w/w), with glycerol as an inducer. On the other hand, Medeiros et al. (2023) found that a higher proportion of wheat bran in the mixture with cottonseed meal (1.5:3.5 w/w) resulted in low lipase activity (0.1 U/g), even with inducers such as olive oil, tilapia oil, Tween 80, and Triton X. Furthermore, Kreling et al. (2021) observed that a higher proportion of wheat bran in the mixture with corncob (80:20) resulted in lower lipase activity (7.77 U/g), even with inducers such as sugarcane molasses and soybean oil. Although some studies indicate improved lipase production with wheat bran mixtures, the literature lacks consensus on whether mixing wheat bran with other beans at the same proportions optimizes lipase production. Therefore, further studies are needed to determine the best conditions for maximizing lipase activity.

Mandari et al. (2020) reported that a 6.66:1.66:1.66 w/w blend of *Prosopis juliflora*, red gram husk, and cottonseed meal yielded lipase activity of 269.87 U/g. In this study, lipase activity was measured using *p*-NPP as the substrate. Ohara et al. (2018) achieved lipase activities of 323 U/g and 319 U/g using cottonseed meal and a mixture of wheat bran and soybean meal as raw materials for lipase production, and determined lipase activity using the fatty acid titration method. These observations indicate that a proper combination of cottonseed meal with other components can optimize enzyme activity, considering the specific contributions of each raw material.

### Synthesis and characterization of the poly (Sty-*co*-DVB)

Poly(Sty-*co*-DVB) has been widely demonstrated to be effective at adsorbing lipases from various sources (Pinto et al. 2014, 2020; Silva et al. 2018; Miguel Júnior et al. 2022). Its high hydrophobicity promotes enzyme immobilization, enabling a higher enzyme loading.

Table 4 summarizes the textural properties of the poly(Sty-*co*-DVB) support, including its specific surface area, pore volume, and average pore diameter. These particles exhibited a high specific surface area and average pore sizes, which can be attributed to the composition of the diluent mixture and the high DVB content in the monomeric phase (Okay 2000; Gokmen and Du Prez 2012). The diluent mixture used as a porogenic agent mainly consisted of n-heptane (80% v/v), and the monomer-to-diluent ratio was 150%. N-heptane is regarded as a non-solvating solvent for polystyrene because of the large difference in Hildebrand solubility parameters between n-heptane and the polymer (Costa et al. 2010). Phase separation between the polymeric matrix and the porogenic mixture likely occurred before gelation. At high dilution, larger agglomerates formed, yielding a structure with average pore sizes spanning mesopores to macropores and a generally smaller internal surface area. This process is known as the χ-induced syneresis mechanism (Okay 2000; Gokmen and Du Prez 2012).

**Table 4:** Data on surface area (S), pore volume (Vp), and pore diameter (D) obtained for poly(Sty-*co*-DVB) support

Tol/n-Hep	S	Vp	D
(% v/v)	(m^2^/g)	(cm^3^/g)	(Ǻ)
20/80	285.8	0.99	204.3

Diluents: *Tol* Toluene and *n-Hep* n-heptane

**Fig. 1:**
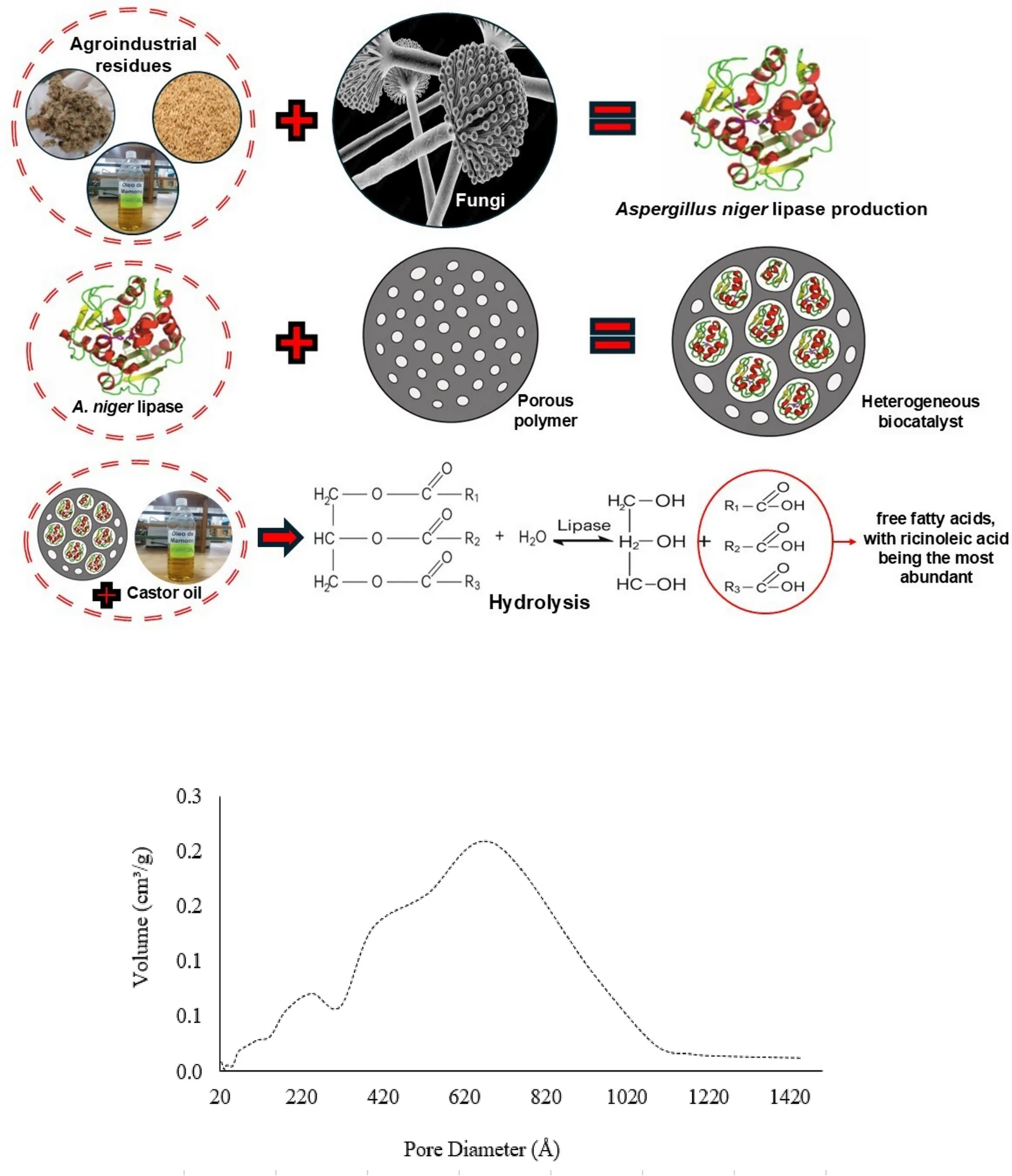
Pore size distribution of Sty-DVB copolymers prepared using a toluene/n-heptane (Tol/n-Hep) porogenic solvent ratio of 20/80 (v/v). The curve shows pore volume (cm^3^/g) as a function of pore diameter (Å)

Figure 1 shows the pore distribution curve for poly(Sty-co-DVB). Notably, a high n-heptane content in the diluent mixture produced a distribution of larger pores. The average pore diameter of this polymer was 204.3 Å (20.43 nm), indicating that this material is mesoporous (Okay 2000).

**Fig. 2:**
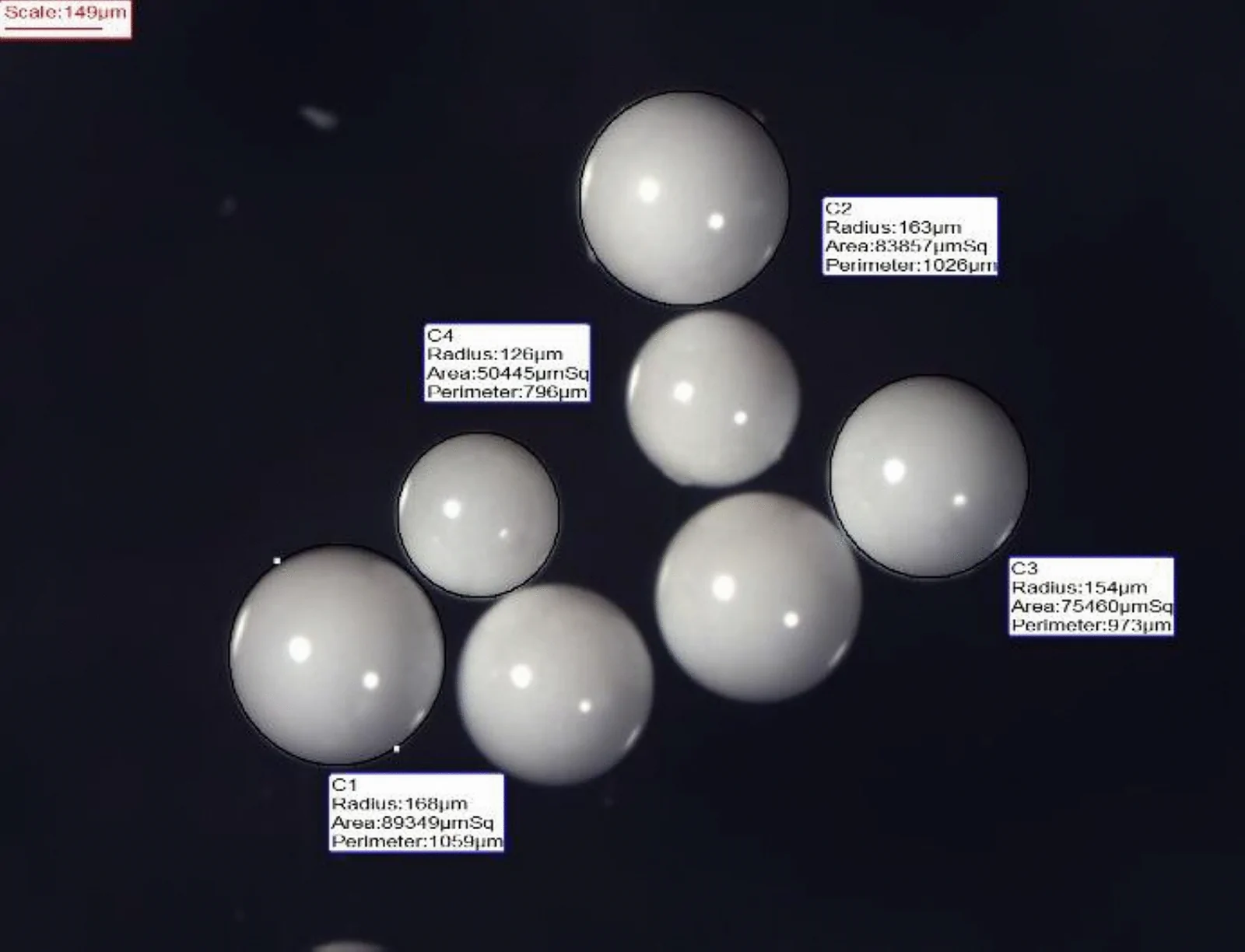
Optical microscopy images of the poly(styrene-co-DVB) copolymer obtained at 4× magnification

**Fig. 3:**
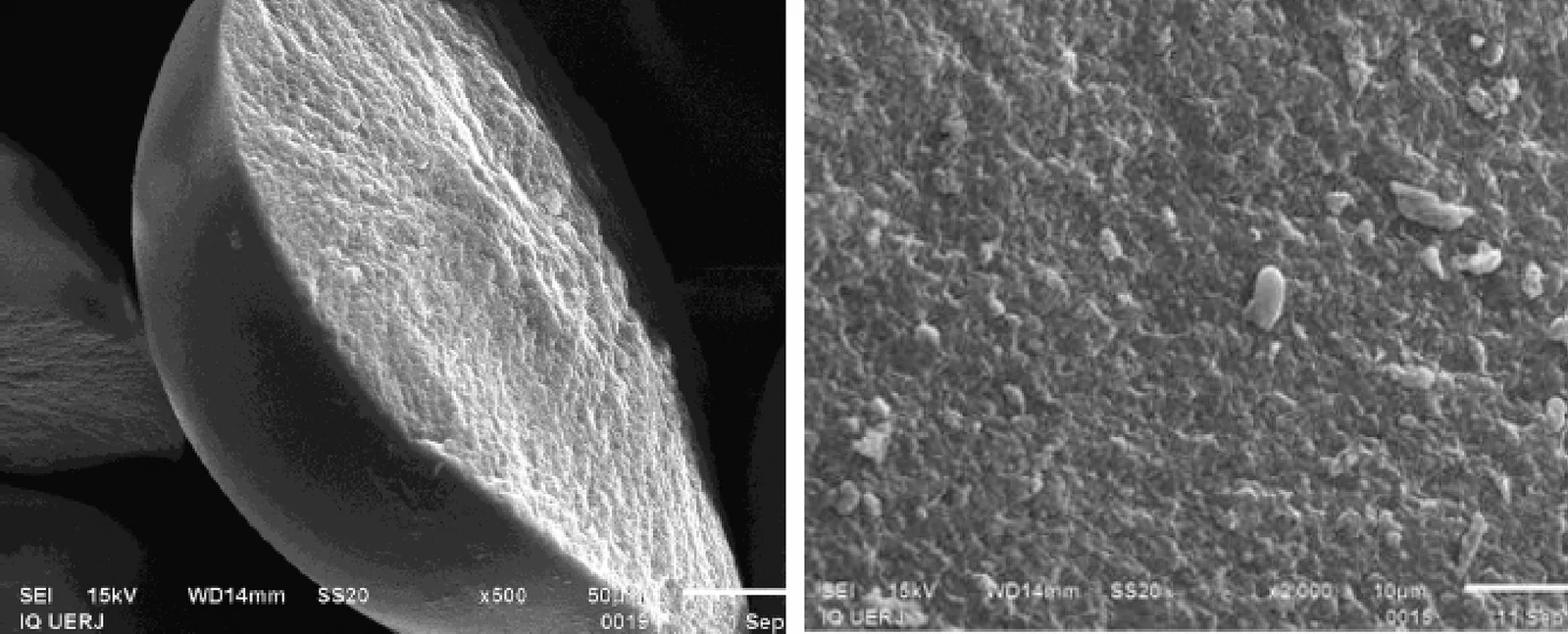
Scanning electron microscopy images of the poly(styrene-co-DVB) copolymer at 500× (left) and 2000× (right) magnifications. Scale bars: 50 μm (left) and 10 μm (right)

In Fig. 2, optical microscopy revealed the formation of particles with individualized structures and spherical, opaque shapes, confirming the presence of porosity. Complementing this analysis, scanning electron microscopy (SEM) enabled a cut to be made in the polymer, allowing observation of its internal structure, which was found to be porous (Fig. 3).

### Immobilization of *Aspergillus niger lipase *to poly(Sty-*co*-DVB)

The lipases extracted from the *A. niger* mutant strain 11T53A14, produced by SSC in experiments 1 to 6 with castor oil supplementation (Table 2), were selected for immobilization studies. These lipases were contacted with poly(Sty-co-DVB) to prepare heterogeneous biocatalysts. A commercial lipase from *A. niger* was used as a control and immobilized in the same copolymer under identical conditions to those used for the lipases produced by SSC.

Immobilization of *A. niger* using SSC produced distinct biocatalysts, with differences in immobilization yield (YI) and recovered activity (Ra) (Table 5). These variations may be due to differences in the protein load applied to the supports, which are intended to standardize activities across the supports.

**Table 5:** Parameters of immobilization to heterogeneous biocatalysts

Biocat	Utheo (U/g_suport_)	YI (%)	R_a_ (%)	HA (U/g_bio_)
Biocat(1)	8.4	67.3	9.4 ± 0.0	0.8 ± 00
Biocat(2)	10.3	82.3	15.0 ± 0.0	1.5 ± 0.0
Biocat(3)	10.7	85.6	30.0 ± 0.0	3.2 ± 0.1
Biocat(4)	10.5	84.0	5.0 ± 0.0	0.5 ± 0.0
Biocat(5)	11.5	92.2	7.5 ± 0.0	0.8 ± 0.2
Biocat(6)	12.3	98.2	17.1± 0.0	2.1± 0.1
Biocat(7)*	12.1	94.9	18.0± 0.0	2.1± 0.1

Biocat(9): obtained employing commercial lipase from *Aspergillus niger*, supplied by Sigma-Aldrich

The biocatalysts derived from lipases (5) and (6), produced from mixtures of bran and castor oil, achieved YI values of 92.2% and 98.2%, respectively, approaching the YI of 94.9% achieved by biocatalyst (7) made with commercial lipase.

Heterogeneous biocatalysts exhibited low hydrolytic activity against the *p*-NPL substrate, ranging from 0.5 ± 0.0 to 3.2 ± 0.1 U/g_biocat_. In addition, the biocatalysts produced in this work showed recovered activities (Ra) ranging from 5 to 30% (Table 5). The low hydrolytic and recovered activities of these biocatalysts can be attributed to mass-transfer limitations, as the substrate must diffuse from the reaction medium into the pores and channels of the support, and the product must diffuse in the opposite direction (Torquato et al. 2025).

The immobilization yield ranged from 67.3% to 98.2%, whereas Ra of the biocatalyst was much lower, ranging from 5 to 30% (Table 5). Ra reflects the proportion of immobilized enzymes that remain active after immobilization. Thus, the low values of this parameter indicate a loss of enzymatic activity during immobilization. Several studies in the literature report biocatalysts with activity recovery below 100% (Pinto et al. 2014, 2020; Cipolatti et al. 2018, 2019; Ribeiro et al. 2022; Fé et al. 2024; Torquato et al. 2025), indicating a recurring behavior across different enzyme immobilization strategies. The loss of enzymatic activity after immobilization can be attributed to changes in enzyme conformation during immobilization, formation of enzymatic dimers, and mass-transfer limitations caused by restricted substrate diffusion through the pores and channels of the solid support (Bolivar et al. 2022).

The biocatalyst derived from lipase obtained in experiment 3 (Table 2), produced using cottonseed meal, showed the highest recovered activity (Ra) of 30% and a hydrolysis activity of 3.2 U/g, significantly surpassing the other lipases produced by SSC and the biocatalyst 7 made using the commercial enzymatic solution. Notably, the lipases obtained by SSC used in the immobilization studies were not purified. This suggests that the cultivation conditions used were effective in producing lipases in the SSC, resulting in characteristics similar to those of commercial lipase, which is marketed in its pure form.

The biocatalyst derived from lipase obtained in experiment 3, produced using pure cottonseed meal and castor oil, showed a lower theoretical adsorbed enzymatic activity (U_theo_) of 8.4 U/_g support_ and an immobilization yield of 67.3%, which is lower than the YI achieved for the other lipases produced. During testing, the enzymatic activity of this biocatalyst was lower than that of the other biocatalysts. Although the cultivation conditions for lipase production were favorable, the immobilization step involves controllable parameters such as pH, temperature, and buffer, which may not directly relate to the lipase fermentation conditions. The variation in protein content in the medium during immobilization was not measured; however, to achieve the same U input during immobilization, a larger amount of protein would be needed, potentially reducing the YI—probably due to the presence of other proteins or enzymes that do not directly contribute to lipolytic activity but were present in the enzyme preparation. The activity of the biocatalysts depends on the balance among the protein load offered, the immobilization yield, and the initial enzyme activity (Fé et al. 2024).

*A. niger*, when grown during the exponential phase, can produce primary metabolites such as proteins and extracellular enzymes, including amylases, pectinases (Rilda et al. 2023), proteases, cellulases (Ohara et al. 2018), oxidases, dehydrogenases, hydrolases (Zhu et al. 2020), and xylanases (Moran-Aguilar et al. 2021). It can also produce other structurally complex proteins or small peptides, such as nucleoside hydrolase and sterol 24-c-methyltransferase (Zhu et al. 2020). It is unclear which proteins were produced alongside lipase in this study, which may have also affected the results. Proteases produced at different concentrations may influence lipase stability and activity. Immobilizing lipases present in the crude enzyme extract presents challenges. However, this strategy also has advantages. Using crude enzyme extract in the immobilization process can reduce the production costs of the heterogeneous biocatalyst (Putri et al. 2020). Enzymes produced during the cultivation stage can be directly immobilized on supports in a “one-pot" approach, without a purification step. The literature has reported immobilizing lipases on hydrophobic supports as a purification strategy for these enzymes. According to Rodrigues et al. (2019), in low-ionic-strength media, lipase immobilization on hydrophobic supports is favored by the interfacial activation mechanism, and this strategy is considered suitable for both immobilization and partial purification of these enzymes.

### Evaluation of immobilized biocatalysts in the hydrolysis reaction of castor oil

The immobilized biocatalysts were tested for the hydrolysis of castor oil. The main fatty acids in castor oil were analyzed by gas chromatography, showing that 3.3% is palmitic acid (16:0), 2.8% is oleic acid (18:1), 2.8% is stearic acid (18:0), and 2.8% is linoleic acid (18:2). Among the free fatty acids in castor oil, ricinoleic acid is the most abundant, making up about 90% (Rathod and Pandit 2009). The most common constituents in castor oil are triglycerides derived from these fatty acids, with the main triglyceride being the derivative of the most abundant fatty acid.

Table 6 compares the hydrolytic efficiency of the heterogeneous biocatalysts biocat(3), biocat(5), and biocat(7) (Table 5) with that of the commercial heterogeneous biocatalysts (Lipozyme TL, 435, and RM) against castor oil. Hydrolysis efficiency is reported as conversion (%) after 24 h of reaction. The table also provides data on the specific surface area and pore diameter of the supports used to prepare these biocatalysts.

**Table 6:** Evaluation of immobilized biocatalysts in the hydrolytic conversion of castor oil

Heterogeneous biocatalysts	Conversion (%) After 24 h	Support	S (m^2^/g)	D (Å)
Biocat(3)	45.0	poly(Sty-*co*-DVB)	285.8	204.3
Biocat(5)	36.0			
Biocat(7)	17.7			
Lipozyme TL IM	91.2	granulated silica	500^a^	300–700^a^
Lipozyme 435	39.6	poly(MMA-*co*-DVB)	130^b^	150^b^
Lipozyme RM	47.0	poly(phenol–formaldehyde) resin	226^c^	100–300

Biocat(3): Heterogeneous biocatalyst based on lipase extracted from *A. niger* derived from cottonseed meal; Biocat(5): Heterogeneous biocatalyst based on lipase extracted from *A. niger* derived from a combination of cottonseed meal and wheat meal; Biocat(7): Heterogeneous biocatalyst based on commercial *A. niger* supplied by Sigma-Aldrich; Lipozyme TL: *Thermomyces lanuginosus* lipase; Lipozyme 435: *Candida antarctica* fraction B lipase; Lipozyme RM: *Rhizomucor miehei* lipase. a: Mokhtar et al. 2020; b: Ortiz et al. 2019; c: Calero et al. 2019

The biocatalysts biocat(3) and biocat(5), obtained from lipases via SSC, showed conversion efficiencies in the hydrolysis of triglycerides in castor oil that were 154% and 103% higher, respectively, than those achieved by biocat(7) from commercial *A. niger* lipase supplied by Sigma-Aldrich.

Among the commercial lipases, Lipozyme TL IM, based on lipase from *Thermomyces lanuginosus*, was the most efficient, achieving a conversion rate of 91.2% in the hydrolysis of castor oil, followed by Lipozyme RM, based on lipase from *Rhizomucor miehei*, with 47.0% efficiency in this reaction. Although the biocatalysts prepared in this work did not surpass the commercial heterogeneous biocatalyst Lipozyme TL, the lipases produced from *A. niger* demonstrated competitive performance, especially biocat(3), derived from lipase from cottonseed meal and castor oil, which showed conversions in castor oil hydrolysis close to those of Lipozyme RM and higher than the efficiency of the biocatalyst Lipozyme 435, the most commonly studied commercial heterogeneous biocatalyst in the literature (Ortiz et al. 2019).

**Fig. 4:**
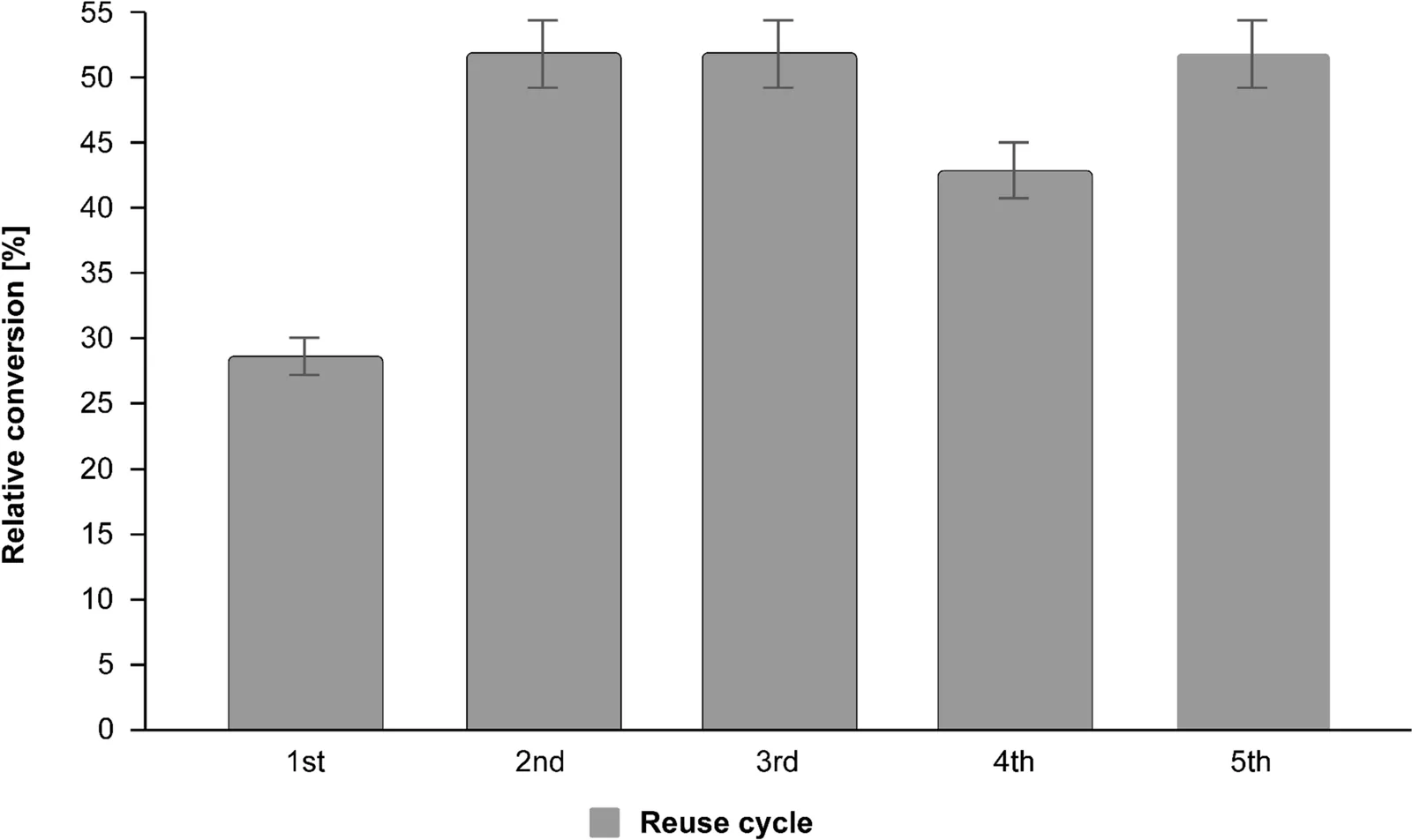
Relative conversion (%) during five reuse cycles of the immobilized biocatalyst. Error bars represent the standard deviation (n = 3)

In our research group, the enzyme-loading conditions used in the present work were previously standardized through independent studies designed to evaluate the relationship between protein loading and catalytic activity. Based on these optimization studies (Cipolatti et al. 2018; Pinto et al. 2019; Fé et al. 2024), a fixed enzyme loading was selected to ensure reproducibility and enable reliable comparisons among the different immobilized preparations. The heterogeneous biocatalysts exhibited varying surface areas and pore sizes, which may account for differences in performance. For instance, Lipozyme TL has a specific surface area of 500 m^2^/g and pore sizes of 300–700 Å, supported on materials such as silica or resins (Mokhtar et al. 2020). Lipozyme 435 has a specific surface area of 130 m^2^/g and pore sizes of 315–1000 Å, typically immobilized on polymethacrylate crosslinked with divinylbenzene (Remonatto et al. 2022). Lipozyme RM has a specific surface area of 226 m^2^/g and pore sizes of 100–300 Å, supported on silica or cellulose esters (Calero et al. 2019). These differences can directly influence substrate diffusion and enzyme accessibility, thereby affecting the final conversion. In addition to hydrolytic efficiency, the operational stability of the biocat(3) was evaluated over 5 consecutive cycles (24 h each) (Fig. 4). A tendency to maintain the conversion percentage throughout was observed. As noted in a previous work, “the reuse of the heterogeneous biocatalyst in the hydrolysis reaction for five complete cycles indicates the possibility of reducing the total cost of the process when applying this biocatalyst in a large-scale application (concerning the free enzyme)” (Torquato et al. 2025).

## Conclusions

The cultivation variables—moisture content, porosity, bran composition, and lipid content—were important in this study, directly affecting lipase production efficiency. The combination of cottonseed meal with castor oil proved to be 1.5 and 1.6 times more efficient (U/g_dry mass_) in lipase production by *A. niger* than the mixture of cottonseed meal and wheat. This increase in efficiency can be attributed to the higher lipid content of cottonseed meal combined with castor oil. *A. niger* lipase obtained from pure cottonseed meal, supplemented with castor oil (2% w/w) and cultivated at 54% initial moisture for 48 h of SSC, resulted in a maximum enzymatic activity of 93 U/g dry mass.

The production of *A. niger* lipases in SSC yielded distinct immobilized biocatalysts, with variations in immobilization yield (RI) and activity retention (Ra). These differences can be attributed to variations in the protein load offered to the support. The biocatalyst derived from *A. niger* lipase produced using a mixture of cottonseed meal, wheat, and castor oil achieved an immobilization yield (IY) of 98.2%, which is higher than the 94.9% IY of the commercial lipase.

The lipase-derived biocatalyst (5), produced from pure cottonseed meal and castor oil, retained 30.0% of its activity (Ra) when measured with *p*-NFL and converted 22.3% of castor oil via hydrolysis. It outperformed the commercial lipase, which had an Ra of 18.0% and a 9.7% conversion, as well as the commercial biocatalyst Novozymes TL, which showed 17.7% conversion during castor oil hydrolysis. Its efficiency in hydrolyzing castor oil was similar to that of the commercial biocatalyst Lipozyme 435, which achieved a 20.3% conversion. However, the commercial biocatalyst Lipozyme RM had the highest conversion rate at 42.4% in this hydrolysis reaction among all immobilized biocatalysts.

The biocatalysts (5) and (8), derived from lipase produced via solid-state cultivation, achieved conversions of 22.3% and 16.3%, respectively, in the hydrolysis of castor oil, surpassing some commercial lipases. Although modest, these results demonstrate the competitiveness of the immobilized biocatalysts and highlight the potential of Sty-DVB polymeric supports. The ability to reuse the immobilized enzyme for 5 cycles in the hydrolysis of castor oil without loss of catalytic activity suggests that this strategy can reduce the overall cost of this reaction when applied on a larger scale.

## Data Availability

The data used to support the findings of this study were included in the article.
